# Description of a new species and subspecies of *Idalus* Walker from Costa Rica, Honduras and Guatemala (Lepidoptera, Erebidae, Arctiinae, Arctiini)

**DOI:** 10.3897/zookeys.264.4403

**Published:** 2013-02-06

**Authors:** Bernardo A. Espinoza, Daniel H. Janzen, Winnie Hallwachs, J. Bolling Sullivan

**Affiliations:** 1 Instituto Nacional de Biodiversidad (INBio), 22-3100 Santo Domingo de Heredia, COSTA RICA; 2 Department of Biology, University of Pennsylvania, Philadelphia, PA 19104, USA; 3 Department of Biology, University of Pennsylvania, Philadelphia, PA 19104, USA; 4 200 Craven Street, Beaufort, North Carolina 28516, USA

**Keywords:** Lepidoptera, Erebidae, Arctiinae, Arctiini, *Idalus paulae* sp. n., *Idalus maesi faustinoi* subsp.* n*., *Idalus maesi* Laguerre, Myrtaceae, Costa Rica, Honduras, Guatemala

## Abstract

A new species and subspecies of *Idalus* Walker are described from Costa Rica, Honduras and Guatemala. Images of males and females and their genitalia are provided. Locality information and distribution maps for Costa Rica and for Guatemala are included. The biology and phylogeny of *Idalus* are discussed.

## Introduction

Walker (1855) described the genus *Idalus* based on the species *Phalaena admirabilis* Cramer from Surinam. Hampson (1901) provided a further description of the genus, and described only one additional species, with Rothschild subsequently describing nearly half the species currently placed in the genus (Watson and Goodger 1986). Travassos (1949) reviewed 19 species, comparing them to *Idalus admirabilis* from Amazonas and Southeastern Brazil as well as from Cayenne, French Guiana. The genus presently includes 50 species from the Neotropical region (Watson and Goodger 1986). Collecting in Costa Rica over the past 20 years has revealed 17 distinct species when compared to types in the British Museum of Natural History and the United States National Museum (B. Espinoza, unpublished). Some of these “species” appear to contain several sibling species as revealed by CO1 barcoding (D. H. Janzen, W. Hallwachs, B. Espinoza, unpublished; Janzen et al. 2009). This paper describes one of the distinct new species and compares it to populations with similar wing patterns found throughout Central America which also required that we name a new subspecies as well. The Arctiinae of our usage is the same as Arctiidae of earlier usage (Fibiger and Lafontaine 2005), with *Idalus* in the tribe Arctiini (Watson and Goodger 1986).

## Materials and methods

Genitalia were prepared following procedures detailed by Lafontaine (2004). Photographs of adults and genitalia were taken with a Nikon CoolPix 4500, using a super micro lens. Samples of five specimens from Costa Rica and 24 from Guatemala were taken for DNA barcoding and sent to the Canadian Center for DNA barcoding in Guelph, Ontario. The resulting nucleotide sequences, a.k.a. “DNA barcodes” were compared with neighbor-joining trees using Kimura 2-parameter distances (methods in Ratnasingham and Herbert 2007).

### Repository abbreviations

**INBio** Instituto Nacional de Biodiversidad, Heredia, Costa Rica

**USNM** Natural Museum of Natural History, Smithsonian Institution, Washing- ton D. C.

**BMNH** The Natural History Museum (formerly, the British Museum of Natural History), London, UK

**MNHN** Museum National d’ Histoire Naturelle, Paris, France

**UVG** Departamento de Biologia de la Universidad del Valle de Guatemala

**JBS** Personal collection of J. Bolling Sullivan, Beaufort, North Carolina

**JMS** Personal collection of Jose Monzon Sierra, Guatemala

**ML** Personal collection of Michel Laguerre, Bordeaux, France

## Systematics

### Idalus paulae

Espinoza sp. n.

urn:lsid:zoobank.org:act:4002FE75-D422-476C-92C1-4C43ED2466B0

http://species-id.net/wiki/Idalus_paulae

Figs 1, 2 , 5 , 7

#### Type material.

**Holotype.** ♂:COSTA RICA: Prov. Heredia, Santa Barbara, Finca La Kandela, 1400–1500m, 10.079°N, 84.159°W, 19–23.Oct.2011, leg. B. Espinoza; Trampa de Luz; Voucher # INB0004301794; GenBank accession # JX681671. [INBio].

#### Paratypes.

9♂5♀ COSTA RICA: 1 ♂, Prov. Cartago, Tapantí, Río Grande de Orosi, 1300–1400m, 9.775°N, 83.796°W, 09.Apr.1984, leg. DH Janzen & W. Hallwachs; Voucher # INB0003455064, (dissected); 1 ♂, Prov. Puntarenas, Las Tablas, P. Internac. La Amistad, 1920 m, 8.949°N, 82.744°W, 13.Apr.1989, leg. G. Mora, M. Ramirez; Voucher # INBIOCRI000014399, (dissected); 1 ♂, Prov. Puntarenas, P.N. Piedras Blancas, Sector Riyito, 100m, 8.736°N, 83.288°W, 10.Sep.2002, leg. H. Mendez; Tp. de Luz; Voucher # INB0003536513; 1 ♂, Prov. S. José, P. N. Braulio Carrillo, Est. Zurqui, 500 m antes del Tunel, 1600m, 10.059°N, 84.012°W, 01.May.1991, leg. G. Maass; Voucher # INBIOCRI000358645; 2 ♂, Prov. Here, Res. Biol. Chompipe, C. Chompipe, 2100m, 10.088°N, 84.071°W, 9.Set.1991, leg. J. F. Corrales; Voucher # INBIOCRI000392944, INBIOCRI000392945; 1 ♂, Prov. Alajuela, La Paz Waterfall Gardens, alrededores del hotel. 1480m, 10.204°N, 84.167°W, 5–10.Aug.2007, leg. B. Espinoza; Tp. de Luz; Voucher # INB0004313093; 1 ♂, Prov. Cartago, La Unión. Z. P. C. Carpintera, Campo Esc. Istarú, 1750m, 9.891°N, 83.971°W, 16–17.Aug.2008, leg. R. Rojas; Tp. Luz Mercurio; Voucher # INB0004160698; GenBank accession # JX681685; 1 ♂, Prov. Heredia, Santa Barbara, Finca La Kandela, 1400–1500m, 10.079°N, 84.159°W, 19-23.Oct.2011, leg. B. Espinoza; Trampa de Luz; Voucher # INB0004301795; GenBank accession # JX681694; 1♂, Prov. San José, San Gerardo de Dota, QERC, 2230m, 9.619°N, 83.835°W, 16–27.Mar.2003, leg. J. B. Sullivan, J.D. Lafontaine; 1♀, Prov. San José, Par. Nac. Braulio Carrillo, Estación Zurquí (el Túnel), 1500m, 10.063°N, 84.011°W, 01.Oct.1985, leg. I. y A. Chacón; Voucher # INB0003506722; 1 ♀, Prov. San José, Par.Nac. Braulio Carrillo Estación Zurquí (el Túnel), 1500m, 10.063°N, 84.011°W, 9–11.Jun.1986, leg. I. y A. Chacón; Voucher # INB0003428272; 1♀, Prov. S. José, P. N. Braulio Carrillo, Est. Zurquí, 500 m antes del Tunel, 1600m, 10.059°N, 84.012°W, 01.May.1991, leg. G. Maass; Voucher # INBIOCRI000654190, (dissected); 1 ♀, Prov. Cartago, Paraíso, Pque Nal Tapantí, Sect La Represa, del Puente del Río Porras 300m SE, 1660m, 9.695°N, 83.781°W, 01.Jul.2002, leg. R. Delgado; Tp de Luz; Voucher # INB0003520571, (dissected); GenBank accession # JX681695. Paratypes deposited in INBio, BMNH, USNM, JBS.

#### Etymology.

This species is named for Ana Paula Zamora Espinoza, the author’s niece who has brought much happiness to her family.

#### Diagnosis.

**Figures 1–4.**
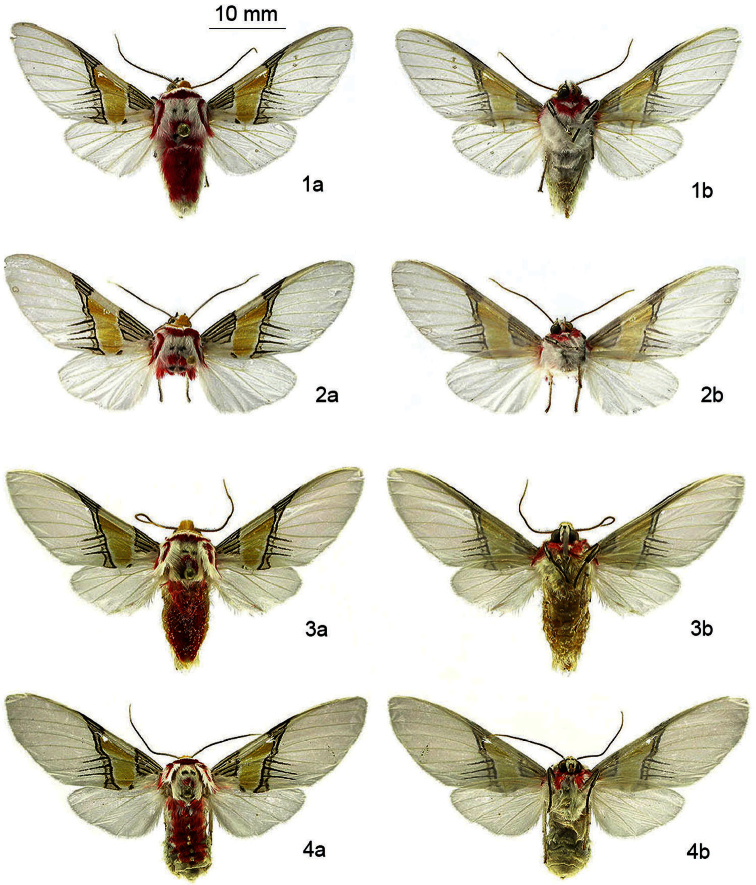
Adults of *Idalus paulae* sp. n. and *Idalus maesi faustinoi sub*sp. n. **a** dorsal view, **b** ventral view. **1a, b** *Idalus paulae*, sp. n., male holotype, INB0004301794 **2a, b** *Idalus paulae* sp. n., female paratype, INB0003520571 **3a, b** *Idalus maesi faustinoi*, subsp. n., male holotype, BAES000004 **4a, b** *Idalus maesi faustinoi*, subsp. n., female, BAES000012

**Figures 5–6.**
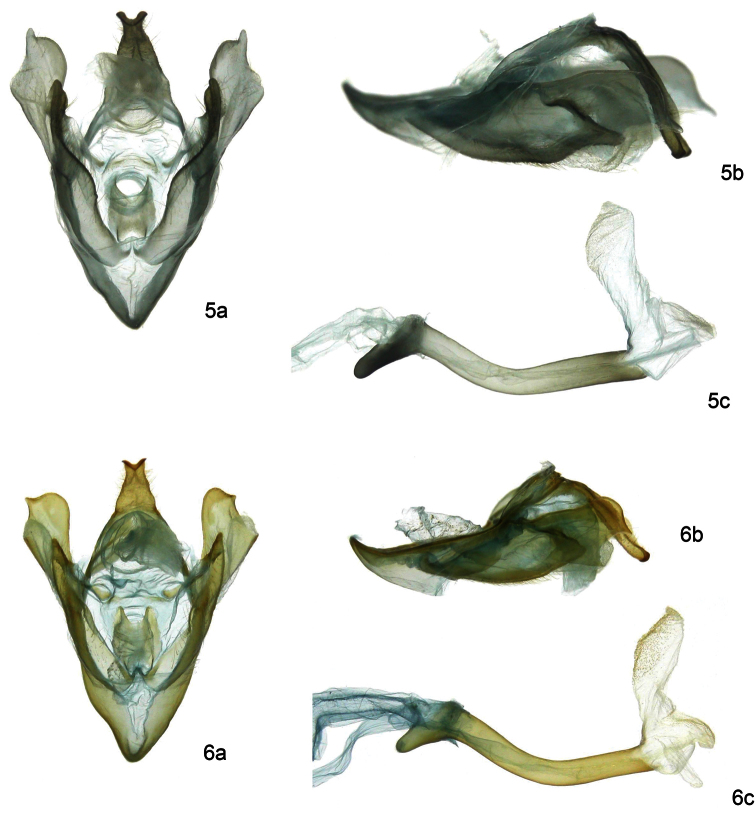
Male genitalia of *Idalus*. **a** ventral view of genital capsule **b** lateral view of genital capsule **c** left lateral view of aedeagus **5a, b, c** *Idalus paulae*, paratype, INBIOCRI000014399 **6a, b, c** *Idalus maesi faustinoi*, paratype, BAES000017

This species can be recognized by the four dark-brown horizontal stripes on the medial area of forewings, between vein Cu1 and the posterior margin together with the yellow medial area (Figs 1, 2), uncus with a bifid and U-shaped terminus in dorsal view, valve with the saccular margin lobulated in the middle (Figs 5a, 5b) and by its distinctive DNA barcode.It is very similar to *Idalus maesi faustinoi*, which has only three dark brown horizontal stripes on postmedial area of forewing.

#### Description.

**Figures 7–8.**
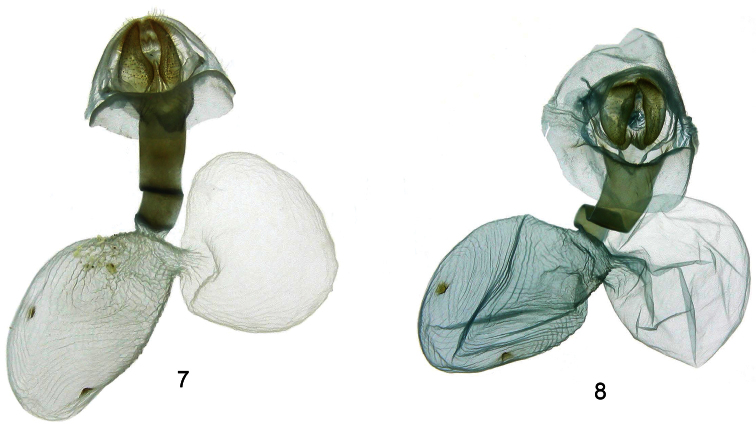
Female genitalia of *Idalus*. **7** *Idalus paulae*, paratype, INB0003520571, ventral view **8** *Idalus maesi faustinoi*, BAES000024, ventral view.

**Adult male** (Figs 1a, 1b, 5a, 5b, 5c). *HEAD*: Small head and large eyes; antennae serrate, base and antennae tip white, mid shaft brown; vertex slightly raised and hairy, yellow-orange; frons white with irregular mesial dark brown patch; labial palpi short and robust, upper half dark brown and lower half white; proboscis well developed. *THORAX*: Patagium white with red posterior edge and transverse yellow-orange band; tegula white, red laterally with a stripe curving inward extending from the base to the apex; thorax robust, white, with two small and elongate dark brown anterior patches, two large, red mid-dorsal patches and two small dark brown rounded patches on the posterior margin, ventrally white, hairy and with a red, longitudinal and ventrolateral stripe below the wings; anterior coxae striped with red. Forelegs white, femur dark brown on the proximal surface, tibia double striped with dark brown from base to tips, tarsal segments brown on the anterior surface; middle legs white, femur with a dark brown patch on tips, tibia double striped with dark brown on the proximal half, tarsi white with brown irregularly on posterior surface; hind legs white, a dark brown patch at the junction of the femur and tibia, tarsi white with irregular brown on posterior surface. *ABDOMEN*: White ground color, dorsally red between terga 1 and 7, basal segment with an irregular patch of long white hair and a series of small white dots between terga 3 and 7; white ventrally. *WINGS*: Forewing length 20.4 mm (*n* = 10). Semihyaline, white ground color, a creamy-white triangular basal patch edged with brown and with four fine dark brown longitudinal lines between the costal margin and the anal vein; a small brown spot in the postbasal area running to the posterior margin; medial area with a large yellow-orange patch between anal margin and R1; a fine transverse dark brown line goes from the anal margin toward the costal margin and turns medially on it; a second parallel fine line, straight from the costal margin to the vein Cu1, then undulating from below it to the anal margin; on the costal margin and between the transverse lines, two very fine dark brown lines between the costal vein and R1 and four more stripes, one between Cu1 and Cu2, two between Cu2 and the anal vein and one between the anal vein and the posterior margin. Hindwing semihyaline white, expanded in humeral area. *GENITALIA* (Figs 5a, b, c): uncus elongate, slightly flattened dorso-ventrally and with an acute shape distally, on its dorsum a thin, longitudinal mesial ridge arising from near the base and almost reaching the tip, terminus bifid and U-shaped in dorsal view and with an acute edge. Valve sclerotized, very wide at the base, acute and slightly concave at the apex and with the saccular margin lobulated in the middle; a large lobe arising distally from the outer surface with a straight costal margin, the saccular margin lobulated in the middle and with a small and acute projection at the tip. Juxta convex, very slightly sclerotized but not well defined; transtilla membranous and very slightly sclerotized at the base of valvae; saccus short and V-shaped; aedeagus long, thin, curved ventrally in the anterior part and curved dorsally in the distal part; vesica short, with two basal diverticuli, one small lateral diverticulum on the right side and another in front projecting slightly ventrally and spinulose, distal portion of vesica with a large spinulose patch. **Adult female** (Figs 2a, 2b, 7). *HEAD*: antennae serrate but less so compared to male antennae; sensillae less dense and shorter in length compared to males. *THORAX*: markings as in male. *ABDOMEN*: markings as in male, but more robust and rounded at the tip. *WINGS*: Slight sexual dimorphism, with the only differences being that the shape and size of the female wing (forewing length**:** 22.8 mm (n = 04)), is broader and longer than that of the male, and the wing apices of the females are more rounded than in the males. *GENITALIA* (Fig. 7): Anal papillae flattened laterally and rectangular in lateral view with a dense patch of short setae dorso-laterally; posterior apophysis 2.5 x as long as anterior apophysis; ostium sclerotized, very wide, dorsal margin rugose, ventral margin deeply concave and U-shaped; ductus bursae elongate, sclerotized and compressed dorsoventrally; corpus bursae oval, membranous and rugose with two small spiculate signa, one on each side the bursa; appendix bursa oval, slightly smaller than corpus bursa.

#### Distribution and biology.

**Figure 9.**
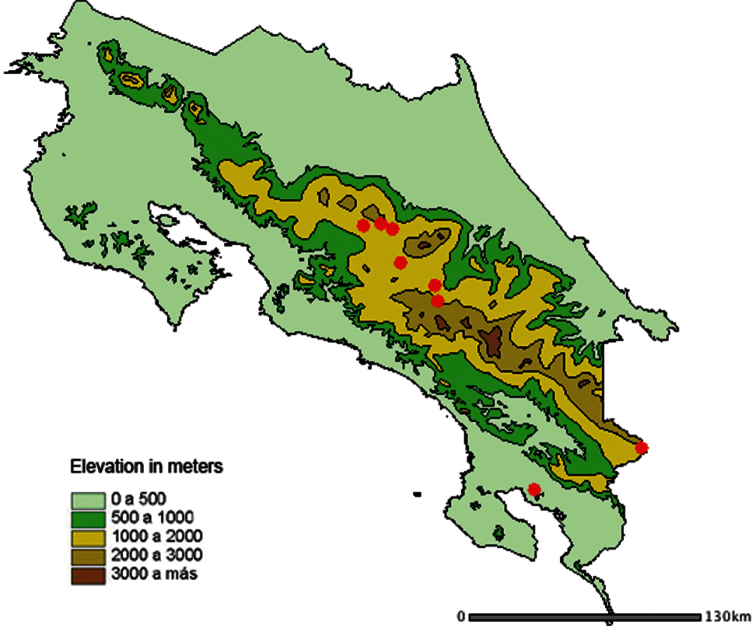
Distribution map of *Idalus paulae* sp. n. in Costa Rica.

*Idalus paulae* has been collected between 1400 and 2230 m elevation in rain forest and the margins of cloud forest, on both Pacific and Atlantic slopes, from 1400 to 2230 m in the Cordillera Volcanica Central and the Cordillera de Talamanca of Costa Rica. There is a single specimen from the rain forest lowlands on the lowest slopes of the Cordillera Talamanca (100 m.) (Fig. 9). This is an unexpected locality but we are unable to find any differences between it and the remaining series of this species. Adults of *Idalus paulae* have been collected throughout the year but only in short series as the species is uncommon at lights and in light traps. No immature stages have been found but *Idalus admirabilis*, a related species, is known to feed on plants of the family Myrtaceae (Porto Santos et al. 2006).

### Idalus maesi faustinoi

Espinoza subsp. n.

http://species-id.net/wiki/Idalus_maesi_faustinoi

Figs 3, 4 , 6 , 8

#### Type material.

**Holotype**. ♂**:** GUATEMALA: Zacapa, San Lorenzo, El Naranjo, 1616m, 15.073°N, 89.685°W, 06.Jan.2010, leg. José Monzón; Voucher # BAES000004; GenBank accession # JX681678. [INBio]. **Paratypes. GUATEMALA:** 3♂, Zacapa, San Lorenzo, El Naranjo, 1616m, 15.073°N, 89.685°W, 06.Jan.2010, leg. José Monzón; Voucher # BAES000001, BAES000002, GenBank accession # JX681669; BAES000003, GenBank accession # JX681672; 7♂1♀, Suchitepequez, Santa Barbara, Ref. Quertzal UVG, 1600m, 14.542°N, 91.197°W, leg. Monzón y Camposeco; 1♂, 2010, Voucher # BAES000013; GenBank accession # JX681670; 1♀, 2010; Voucher # BAES000016; GenBank accession # JX681693; 1♂, 8.Feb.10, Voucher # BAES000014; GenBank accession # JX681691 (dissected); 5♂, 13.Jan.10, Voucher # BAES000017, GenBank accession # JX681681 (dissected); BAES000019, GenBank accession # JX681675 (dissected), BAES000020, GenBank accession # JX681677, BAES000021, GenBank accession # JX681687; BAES000018, GenBank accession # JX681667; 4♂2♀, San Marcos, Camino Fraternidad a Bojonal, 1600m, 14.946°N, 91.881°W, 12.Apr.2010, leg. José Monzón S.; 4♂, Voucher # BAES000007, GenBank accession # JX681698; BAES000008, GenBank accession # JX681683; BAES000009, GenBank accession # JX681665; BAES000010, GenBank accession # JX681676; 2♀, Voucher # BAES000011, GenBank accession # JX681674; BAES000012, GenBank accession # JX681682; 2♂, Huehuetenango, Barillas Unión Las Palmas, 1444m, 15.931°N, 91.299°W, 12.Apr.10, leg. Camposeco y Monzón; Voucher # BAES000005, GenBank accession # JX681689 (dissected); BAES000006, GenBank accession # JX681668. **Additional material examined.** **HONDURAS**: 1♀, Tegucigalpa, Cloud forest, 7000ft, 8.Aug.1972, leg. Robert D. Lehman; Black light (dissected). **GUATEMALA:** 9♂2♀, Suchitepequez, Santa Barbara Ref. Quertzal UVG, 1600m, 14.542°N, 91.197°W, leg. Monzón y Camposeco. 1♂, 13.Mar.2010, Voucher # BAES000022, GenBank accession # JX681690; 2♀, 13.Mar.2010, Voucher # BAES000023, GenBank accession # JX681696; BAES000024, GenBank accession # JX681697 (dissected); 1♂, 10.Apr.2010, Voucher # BAES000015, GenBank accession # JX681686; 2 ♂, 10.Sep.2007; 1♂, 01.Oct.2008; 1♂, 20.Sep.2009; 1♂, 15.Nov.2009; 2♂, 12.Dec.2009; 5♂2♀, San Marcos, Camino Fraternidad a Bojonal, 1600m, 14.946°N, 91.881°W, leg. José Monzón S. 2♂, 1♀, 27.Jul.2008; 1♀, 8.Aug.2008; 1♂, 6.Oct.2008; 2♂, 22.Sep.2009; 1♂, Huehuetenango, Barillas Unión Las Palmas, 1444m, 15.931°N, 91.299°W, 03.Sep.2010, leg. Camposeco y Monzón; 2♂, Baja Verapaz, Cerca Purulha, Hotel Ranchitos del Quetzal, 1656m, 13.Jul.2009, 15.216°N, 90.219°W, leg. Col. José Monzón S.; 1♀, Suchitepequez, Atitlan Reserve, 1570m, 14.328°N, 91.116°W, 9–10.May.2007, leg. M. Laguerre; Voucher # MILA.0407; Dissected, Gen. Voucher # MILA 1784; 1♂, Sacatepequez, San Cristobal el Bajo, Finca El Pilar, 1960m, 14.323°N, 90.422°W, 24.Jul.2004, leg. J.Haxaire & O.Paquit. Paratypes deposited in INBio, BMNH, USNH, UVG, JMS, ML, MNHN.

#### Etymology.

This subspecies is named after Faustino René Camposeco López, a field technician of the Universidad del Valle de Guatemala, who with José Monzón helped collect the type series.

#### Diagnosis.

This subspecies is closely related to *Idalus paulae* which has four dark-brown horizontal stripes on postmedial area of forewings but can be distinguished by the three dark brown horizontal stripes distal to the yellow median area, between vein Cu1 and the anal vein (Figs 3, 4), uncus with a bifid and V-shaped terminus in dorsal view, valve with the saccular margin very slightly lobulated in the middle (Figs 6a, 6b) and by its distinctive DNA barcode. From *Idalus maesi maesi* it is easily recognized by it maculation patterns, in *Idalus maesi maesi* the basal and medial area of forewings, as well as patagium are white, the thorax has no red mid-dorsal patches and the basal abdominal segments are white. DNA barcode of *Idalus maesi maesi* and *Idalus maesi faustinoi* differ by 0.65%.

#### Description.

**Adult male** (Figs 3a, 3b, 6a, 6b, 6c). *HEAD*: Head small and eyes large; antennae serrate, base and antenna tip white, mid shaft brown; vertex yellow-orange, slightly raised and hairy; frons white with irregular mesial dark brown patch; labial palpi short and robust, upper half dark brown and lower half white; proboscis well developed; *THORAX*: Patagium white with a red posterior edge and transverse yellow-orange band; tegula white, red laterally and with a stripe curving inward extending from the base to near the apex; thorax robust, white, with two small, elongated dark brown anterior patches, two large, red mid-dorsal patches and two small dark brown rounded patches on the posterior margin, ventrally white, hairy and with a red, longitudinal and ventrolateral stripe below the wings; anterior coxae striped with red. Forelegs white, femur dark brown on the proximal surface, tibia double striped with dark brown from base to tips, tarsal segments brown on the anterior surface; middle legs white, femur with a dark brown patch on tips, tibia double striped with dark brown on the proximal half and with a small dark brown patch on tips, tarsi white with brown irregularly on posterior surface; hind legs white, a dark brown patch at the junction of the femur and tibia and at the junction of the tibia with the tarsi, tarsi white with irregular brown on posterior surface. *ABDOMEN*: White ground color, dorsally red between terga 1 and 7, basal segment with an irregular patch of long white hair and a series of small white dots between terga 3 and 7; white ventrally. *WINGS*: Forewinglength 19.2 mm (n = 19). Semihyaline, white ground color, a creamy-white triangular basal patch edged with brown and with four fine dark brown longitudinal lines between the costal margin and the anal vein; a small brown spot in the postbasal area running to the posterior margin; medial area with a large yellow-orange patch between anal margin and R1; in the medial area, a fine transverse dark brown line goes from the anal margin toward the costal margin and turning medially on it; a second parallel fine line, straight from costal margin to the vein Cu1 and then undulating from below it to the anal margin; on the costal margin and between the transverse lines, two very fine dark brown lines between the costal vein and R1 and three more stripes, one between Cu1 and Cu2 and two between Cu2 and the anal vein. Hindwing semihyaline white, expanded in humeral area. *GENITALIA* (Figs 6a, b, c): uncus elongate, slightly flattened dorso-ventrally and acute distally, on its dorsum a thin, longitudinal mesial ridge arising from near the base and almost reaching the tip, terminus bifid and V-shaped in dorsal view and with acute edges. Valve sclerotized, very wide at the base, acute and slightly concave at the apex and the saccular margin very slightly lobulated in the middle; a large lobe arising distally from the outer surface with a straight costal margin, the saccular margin lobulated in the middle and with a very small, acute projection at the tip. Juxta convex, very slightly sclerotized but not well defined; transtilla membranous and very slightly sclerotized at the base of valvae; saccus short and V-shaped; aedeagus long, thin, curved downward ventrally in the anterior portion and upward dorsally in the distal part; vesica short, with two basal diverticuli, one small lateral diverticulum on the right side and another in front projecting slightly ventrally and spinulose, distal portion of the vesica with a large spinulose patch. **Adult female** (Figs 4a, 4b, 8). *HEAD*: antennae serrate but less so compared to male antennae; sensillae less dense and shorter in length compared to males. *THORAX*: markings as in male. *ABDOMEN*: markings as in male, but more robust and rounded at the tip. *WINGS*: Slight sexual dimorphism, with the only differences being that the shape and size of the female wing (forewing length**:** 22.2 mm (n = 5)), is broader and longer than that of the male, and the wing apices of the females are more rounded than in the males. *GENITALIA* (Fig. 8): Anal papillae flattened laterally and rectangular in lateral view with a dense patch of short setae dorso-laterally; posterior apophysis 2.5 x as long as anterior apophysis; ostium sclerotized, very wide, dorsal margin rugose, ventral margin deeply concave and V-shaped; ductus bursae elongate, sclerotized and compressed dorsoventrally; corpus bursae oval, membranous and rugose with two small spiculate signa, one on each side the bursa; appendix bursa oval, same size as corpus bursa.

#### Distribution and biology.

**Figure 10.**
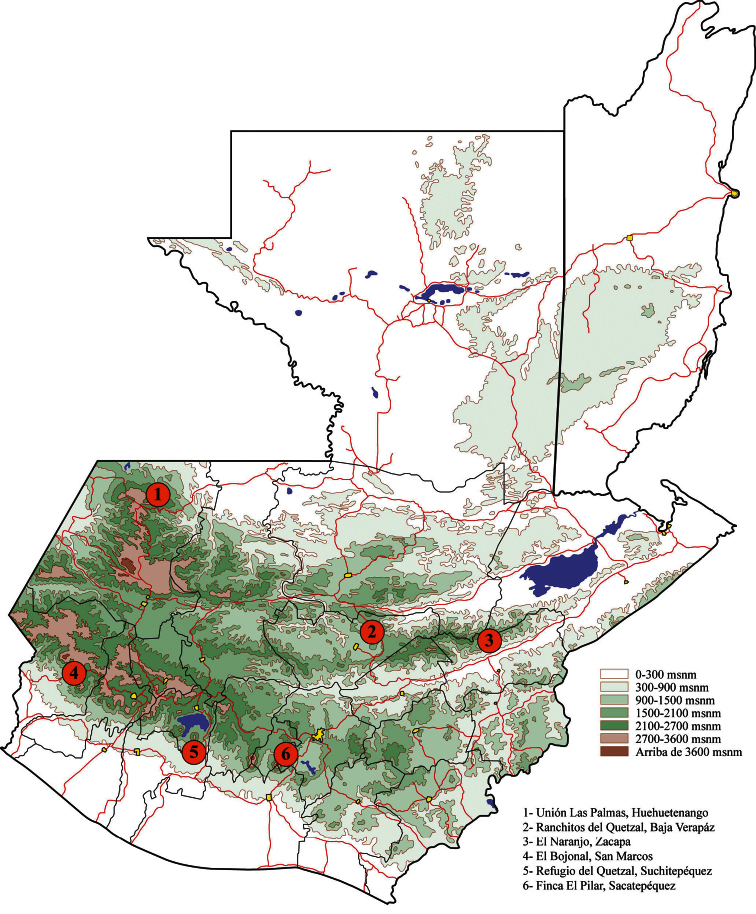
Distribution map of *Idalus maesi faustinoi*, subsp. n. in Guatemala.

In Guatemala (Fig. 10) *Idalus maesi faustinoi* is reported from cloud forest at 1444 to 1616 m elevation (José Monzón personal comm.). A single female specimen from Honduras, Tegucigalpa is recorded from cloud forest at 2133 m. Adults have been captured throughout the year. No immature stages have been found but *Idalus admirabilis*, a related species, is known to feed on Myrtaceae (Porto Santos et al. 2006).

#### Comments.

The Arctiinae (formerly Arctiidae) are estimated to have 11,000 species worldwide, with 6,000 species in the Neotropics (Watson and Goodger 1986). Some 20 years ago the Costa Rican fauna was estimated to be 350 species in 80 genera (INBio collections). At present there are an estimated 1,000 species in 215 genera in Costa Rica (INBio collections), which is remarkable given the small size of the country. Comparative DNA sequencing of the CO1 locus indicates that even more species are present (D. H. Janzen, W. Hallwachs, B. Espinoza, J. B. Sullivan, unpublished data).

According to Travassos (1949, 1950) *Idalus* species have a well-developed proboscis; short and upright labial palpi with the third segment tiny; antenna serrate in both sexes; elongate wings; forewing venation with Sc ending before the apex of wing, R1 arising from discal cell and ending before reaching the apex of wing, R2 and R3 ending before the apex of wing, R4 ending on the apex, R5 ending after apex, M1 arising from anterior angle of discal cell, M2 and M3 arising from posterior angle of discal cell, Cu1 arising from near the posterior angle of discal cell, Cu2 arising from the distal third of the cell, anal vein ending on tornus; hindwing venation with Sc arising from the distal half of cell and not reaching the costal margin of wing, R arising from anterior angle of cell, M and Cu1 arising from posterior angle of cell, Cu2 arising from the distal half of cell, A1 ending before reaching tornus and A2 ending on tornus. The newly described taxa *Idalus paulae* and *Idalus maesi faustinoi* are consistent with these characters. DNA barcoding and neighbor-joining analyses (Ratnasingham and Hebert 2007) of almost all the species of *Idalus* in Costa Rica (*Idalus aleteria* (Schaus), *Idalus carinosa* Schaus, *Idalus crinis* Druce, *critheis* Druce, *Idalus dares* Druce, *fasciipuncta* Rothschild, *Idalus herois* Schaus, *Idalus perlineosa* Rothschild, *Idalus tuisiana* Schaus, *Idalus tybris* (Cramer), *Idalus veneta* Dognin, *Idalus borealis* (Rothschild), *Idalus vitrea* (Cramer), *Idalus vitreoides* (Rothschild), plus several unnamed species) group them in two groups that appear to be related groups. *Idalus paulae*, *Idalus maesi maesi* (Laguerre, 2006) and *Idalus maesi faustinoi* form a related group to *Idalus admirabilis*, *Idalus crinis* and *Idalus herois*. The caterpillars of this latter group of three species is known to eat foliage of Myrtaceae (Porto Santos et al. 2006), (D. H. Janzen and W. Hallwachs, unpublished). The *admirabilis-maesi* groups make up one of the primary branches of *Idalus*. The other primary branch divides into a *vitrea* group and a *perlineosa* group. *Idalus borealis* is in the *vitrea* group and caterpillars have been found feeding on Combretaceae (*Combretum*) (D. H. Janzen and W. Hallwachs, unpublished). *Idalus perlineosa* and a likely undescribed sibling species both in the p*erlineosa* group feed on Proteaceae (*Roupala*) (D. H. Janzen and W. Hallwachs, unpublished). Interestingly, while maculation patterns vary widely in the genus, these patterns unlike the foodplant associations, do not cluster cleanly among the four groups. The color patterns included by Travassos in his *admirabilis* group sort into all four barcode groups.

*Idalus paulae* differs from *Idalus maesi* maesi and *Idalus maesi faustinoi* in DNA sequence of the CO1 locus by approximately 5%. *Idalus maesi maesi* and *Idalus maesi faustinoi* differ by at least 1% but *Idalus maesi faustinoi* breaks into two groups differing by 0.65%. *Idalus maesi maesi* clearly differs from *Idalus paulae* and *Idalus maesi faustinoi* in maculation (the median yellow area is white) but does not appear to differ in male and female genitalia (M. Laguerre, personal communication). Geographically, *Idalus maesi maesi* occurs in the mountains of northern and southern Pacific Nicaragua, *Idalus paulae* in the mountains of Costa Rica, and *Idalus maesi faustinoi* in the mountains of Honduras and Guatemala. The two distinct haplotype groupings of *Idalus maesi faustinoi* may represent two taxa but because they seem to have identical maculation and genitalia and because they do not sort geographically, we have chosen not to name both groups. Instead, the holotype and paratypes are based on populations from the northern montane slopes in Guatemala. It will take additional studies to resolve the haplotype distributions and to establish the food plants upon which this group feeds.

We have not tried to establish morphological characters that separate the *maesi* group from the other three *Idalus* groups. However, we have noticed that in the *maesi* group, sexual modifications of scales and venation in the area of wing overlap are absent but present in those species we have examined from the other three groups. The humeral area is lobed but that character applies to most Arctiinae. The small differences in the CO1 sequences between *Idalus maesi maesi* and *Idalus maesi faustinoi* may reflect recent and rapid evolution of these species in the complex montane regions of Central America.

## Supplementary Material

XML Treatment for Idalus paulae

XML Treatment for Idalus maesi faustinoi
